# Complexation of Boronic Acid with Chiral α-Hydroxycarboxylic Acids and the Ability of the Complexes to Catalyze α-Hydroxycarboxylic Acid Esterification

**DOI:** 10.3390/molecules29010043

**Published:** 2023-12-20

**Authors:** Zhonglei Meng, Rongxiu Qin, Rusi Wen, Junkang Xie, Haiyan Chen, Guiqing Li

**Affiliations:** Guangxi Key Laboratory of Superior Timber Trees Resource Cultivation, Guangxi Forestry Research Institute, Nanning 530002, China; meng-zhonglei@163.com (Z.M.); qrx20151112@126.com (R.Q.); luse-rose@163.com (R.W.); junkangxie@163.com (J.X.); chunjin2005@163.com (H.C.)

**Keywords:** boronic acid, α-hydroxycarboxylic acid, complex configuration, optical rotation, esterification of α-hydroxycarboxylic acid

## Abstract

The complexation of boric acid (BA) with various α-hydroxycarboxylic acids (HCAs) was examined by analyzing the change in the optical rotation after the addition of BA to aqueous HCA solutions, and the catalytic properties of the complexes were examined by catalyzing the esterification of the HCAs. The absolute values of the optical rotation of the HCAs increased with increasing BA-to-HCA molar ratio, and the rate of change of the optical rotation gradually decreased as the BA-to-HCA molar ratio increased, reaching a minimum value at a molar ratio of approximately three. As a catalyst, BA could catalyze the acetylation of hydroxyl groups in addition to the esterification of HCAs. Compared to the conventional synthesis routes of ATBC and ATOC, a synthesis route with BA as the catalyst allowed for a lower frequency of catalyst separation and replacement while providing light-colored products. BA could catalyze the formation of triethyl citrate, and the yield of triethyl citrate reached 93.8%. BA could also catalyze the reaction between malic acid and pinene to produce borneol malate. After saponification of borneol malate, borneol was obtained with a yield of 39%.

## 1. Introduction

**Figure 1:**
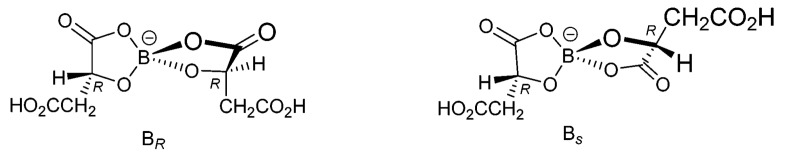
Two borate anions that can form using D-(+)-malic acid [10].

α-Hydroxycarboxylic acids (HCAs) are renewable starting materials with important industrial applications. Studies have reported on the use of tributyl citrate (TBC), acetyl tributyl citrate (ATBC), trioctyl citrate (TOC), and acetyl trioctyl citrate (ATOC) as environmentally friendly plasticizers [1]. Elemental boron is widely distributed on Earth and serves as an indispensable trace element for plants and animals [2,3]. Due to the electrophilicity of boron atoms, boric acid (BA) can form various complexes with hydroxyl-containing compounds, such as single-ligand (i.e., 1:1) and double-ligand (i.e., 1:2) complexes with HCAs [4]. The electrical conductivity and optical rotation increase when tartaric acid forms complexes with boric acid (BA) [5,6]. Larsson et al. [7] analyzed the infrared spectral characteristics of complexes formed by lactic acid and BA in aqueous solution and reported a single-ligand complex formed when pH = 2. Two types of complexes were found to form at a pH of 6, namely single-ligand and double-ligand complexes. Pizer et al. [8] studied the reaction mechanism and chemical equilibrium of the complexes formed by BA and lactic acid, following the temperature increase and the stop flow methods. The researchers reported the ligand in the single-ligand complex inhibited the coordination of the second ligand. Maseda et al. [9] studied the reaction thermodynamics of complexation reactions of BA or borate with hydroxy acid or diols and found the reactions involving hydroxy acid and glycol for the formation of single-ligand complexes were driven by enthalpy. During the formation of a double-ligand complex, the process involving hydroxy acid was driven by an increase in entropy, while the process involving the diol was driven by enthalpy. Marziyeh et al. [10] transformed cloisite 30B (CL) into tosylated cloisite (TCL) as a new hetero-functional support. Lipase from *Candida rugosa* was covalently immobilized on TCL (TCLL) and used to produce biodiesel from waste frying oil (WFO). Hamidreza et al. [11] prepared epoxy-activated cloisite (ECL) as a new hetero-functional carrier through the reaction between cloisite 30B (CL) and epichlorohydrin and utilized it for the covalent immobilization of lipase from *C. rugosa*. Marin et al. [12] synthesized free radical, cationic salts in trichloroethane containing 18-crown ether using malic acid and potassium borate as the raw materials, where the electrocrystallization process was followed for the reactions. Boron presents two configurations, R and S, as shown in Figure 1, when malic acid forms a double-ligand complex with BA [12].

Various applications have been developed for BA–HCA complexes. Complexes formed by chiral carbon-containing α-hydroxycarboxylic esters and BA have been employed for the isolation of racemic chiral compounds with hydroxyl groups on the chiral carbon [13,14,15,16,17,18,19,20]. Because HCAs can form complexes with BA, loading HCAs on ion exchange resins, activated carbon, or carbon nanotubes can improve their adsorption capacity for BA in wastewater [21,22,23]. The esterification of curcumin with HCAs and BA complexes can also improve the thermal stability of curcumin [24]. As a result, citric acid-BA complexes have been used to improve the dimensional and thermal stability of bamboo materials [25]. Composite catalysts composed of BA and HCA were found to exert a good synergistic catalytic effect during the catalysis of the esterification reaction of camphene and the hydration reaction of pinene [26,27]. In a previous study [28], we analyzed and measured the effects of complexation reactions between BA and HCAs on the ionization equilibrium of the HCAs. Eight HCAs were selected to measure the pH changes in aqueous HCA solutions after BA addition [28]. We found the composition of the HCA and BA solution had a significant impact on the performance of the catalyst.

Therefore, it was important to study the types of complexes formed with different ratios of HCA to BA; however, due to the complexity of their complexation reactions, measuring the changes in pH alone was not sufficient. Considering the complexity of the reaction between BA and HCA, we hypothesized single-ligand complexes and double-ligand complexes would have different effects on the changes in optical rotation. L-(+)-tartaric acid (L-TA), D-(−)-tartaric acid ester (D-TA), (R)-(−)-mandelic acid (R-MA), L-(−)-malic acid (L-H_2_Mi), D-gluconic acid (D-GlcA), and D-(−)-lactic acid (D-LA) were used to conduct the experiments in this study. The changes in the optical rotation of the compounds after the addition of BA to the system were analyzed, and the catalytic performance of BA-HCA complexes for esterification with HCAs was studied, to obtain scientific data on the effect of the complexes on the changes in rotation and catalytic performance.

## 2. Results and Discussion

### 2.1. Effect of BA on the Optical Rotation of Aqueous HCA Solutions

**Figure 2:**
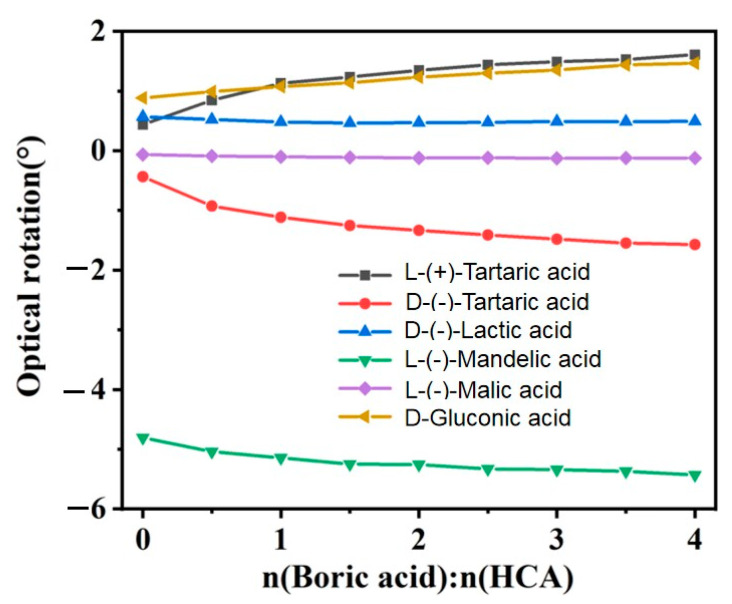
Effect of BA on the optical rotations of various chiral α-hydroxycarboxylic acids (HCAs) at 0.2 mol/kg.

In our previous work, we measured the pH changes of boric acid after adding HCA solution and analyzed the influence of the boric acid and HCA complex reaction on the ionization equilibrium of HCAs. We found the more complex species that formed, the greater the influence on HCA ionization. Thus, the greater the number of hydroxyl and carboxyl groups, the greater the rate of pH change. However, we also found when mandelic acid, lactic acid, and glycolic acid contained the same number of hydroxyl and carboxylic groups, their pH change rates successively decreased [28]. However, lactic acid only contains one more methyl group than glycolic acid, which cannot explain why the changes in pH are at different rates. We hypothesize, in addition to being single- or double-ligand complexes, the three-dimensional configuration of the complex also affects the pH change rate. When a chiral HCA is complexed with boric acid, the polarized light can change due to the formation of a new three-dimensional structure. To test this hypothesis and gain insights into the effects of the molecular structures of HCAs on the BA–HCA complexes, we measured the optical rotations of aqueous solutions of HCAs with BA added at various molar ratios. In this study, BA was added at various BA-to-HCA molar ratios while maintaining the HCA concentration fixed at 0.2 mol/kg. The changes in optical rotation were measured (Figure 2). At 25 °C, the solubility of BA in water was 5.74 g, i.e., S = 5.74 g/100 g water or 0.928 mol/kg. For the aqueous HCA solution with a concentration of 0.2 mol/kg, the BA-to-HCA molar ratio was chosen to be less than 4.6. However, when the BA-to-HCA molar ratio was 4.5, the measured optical rotation fluctuated significantly due to the near-saturation of BA. Therefore, the BA-to-HCA molar ratio was varied from 0 to 4 in the experiment.

As shown in Figure 2, the absolute values of the optical rotation of L(+)-tartaric acid, D(-)-tartaric acid, R-MA, L-H_2_Mi, and D-GlcA increase with increasing BA-to-HCA molar ratio, while the absolute values of the optical rotation of D-LA initially decrease and then increase. Because the groups connected to the chiral carbon of lactic acid consisted of H and CH_3_, the configuration of a small amount of lactic acid was possibly reversed when the complex formed. When the molecule of lactic acid was small, it could easily form 1:2 complexes, resulting in reduced optical rotation. However, with an increase in the molar ratio of boric acid to lactic acid, the amount of rotation of single-ligand complexes gradually increased; thus, the rotation increased after a certain decrease. Lactic acid and boric acid possibly had more influence on the ionization equilibrium than glycolic acid due to an increase in three-dimensional configuration. The catalytic effect of lactic acid is also better than glycolic acid when catalyzing the pinene hydration reaction, as shown in reference [29].

The optical rotations of the HCAs at a fixed molar concentration of 0.2 mol/kg with varying molar concentrations of added BA are fitted, as shown in Figure 2, yielding the following equations:

(1) $$\begin{array}{l} y\;(D-\mathrm{LA})=\;-0.0069x^{3}+\;0.0577x^{2}\;-\;0.1404x+\;0.5729\\ R^{2}=\;0.9799 \end{array}$$

(2) $$\begin{array}{l} y\;(R-\mathrm{MA})=\;-0.0209x^{3}+\;0.1662x^{2}\;-\;0.4841x\;-\;4.8158\\ R^{2}=\;0.9978 \end{array}$$

(3) $$\begin{array}{l} y\;(D-\mathrm{GlcA})=\;-0.0003x^{3}\;-\;0.0097x^{2}+\;0.1898x+\;0.8887\\ R^{2}=\;0.9977 \end{array}$$

(4) $$\begin{array}{l} {y(L-H}_{2}\mathrm{Mi})\;=\;-0.0011x^{3}+\;0.0128x^{2}\;-\;0.0475x\;-\;0.0666\\ R^{2}=\;0.9892 \end{array}$$

(5) $$\begin{array}{l} y\;(L-\mathrm{TA})=\;0.0339x^{{}_{3}}\;-\;0.2874x^{2}+\;0.8986x+\;0.4504\\ R^{2}=\;0.9971 \end{array}$$

(6) $$\begin{array}{l} y\;(D-\mathrm{TA})=\;-0.0362x^{3}+\;0.3001x^{2}\;-\;0.9028x\;-\;0.4744\\ R^{2}=\;0.9896 \end{array}$$

where y is the optical rotation, *x* is the BA-to-HCA molar ratio, and R is the correlation coefficient.

By differentiating Equations (1)–(6), the equations relating the optical rotation rate of change of the 0.2 mol/kg HCAs to the molar concentration of added BA were obtained as follows:

(7) $$\frac{dy(D-LA)}{dx}=-0.0207x^{2}+0.1154x-0.1404$$

(8) $$\frac{dy(R-MA)}{dx}=-0.0627x^{2}+0.3324x-0.4841$$

(9) $$\frac{dy(D-GlcA)}{dx}=-0.0009x^{2}-0.0194x+0.1898$$

(10) $$\frac{dy(H_{2}Mi)}{dx}=-0.0033x^{2}+0.0256x-0.0475$$

(11) $$\frac{dy(L-TA)}{dx}=0.1017x^{2}-0.5748x+0.8986$$

(12) $$\frac{dy(D-TA)}{dx}=-0.1086x^{2}+0.6002x+0.9028$$

where y is the optical rotation for a specified amount of HCA, *x* is the BA-to-HCA molar ratio, and $\frac{dy}{dx}$ is the rate of change of the optical rotation with respect to the molar concentration of added BA.

**Figure 3:**
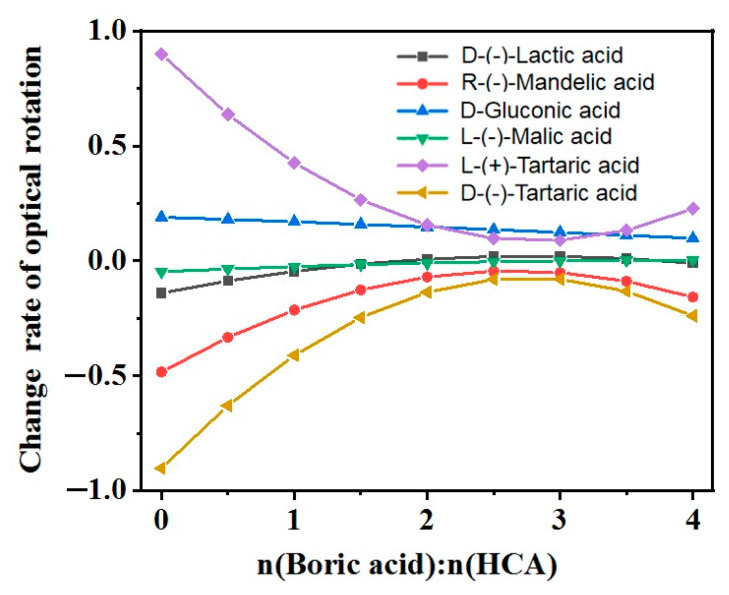
Rates of change for the optical rotations of various chiral HCAs with BA content.

By plotting the data obtained from Equations (7)–(12), the rate of change of the optical rotation of the HCAs with respect to the molar concentration of added BA is obtained, as shown in Figure 3. Figure 3 also shows D-GlcA exhibited a continuous decrease in absolute value of the rate of change of the optical rotation, while the other five chiral HCAs gradually decreased in absolute value before slightly increasing. The BA-to-HCA molar ratios corresponding to the vertices of the curves in Figure 3 were 2.8 for D-LA, 2.7 for R-MA, 10.8 for D-GlcA, 3.9 for L-H_2_Mi, 2.8 for L-TA, and 2.8 for D-(−)-TA. Because the BA-to-HCA molar ratios are positive, the rates of change of the optical rotation of gluconic acid with respect to the molar concentration of added BA are continuously decreasing, as shown in the plot. The rate of change values for the optical rotations of lactic acid and L-H_2_Mi were zero at certain BA-to-HCA ratios, which were 1.8 and 3.8 for lactic acid and 3.1 and 4.7 for L-H_2_Mi.

### 2.2. Comparison of the Effects of Protonic Acid and BA on the Optical Rotation of Chiral HCAs

**Figure 4:**
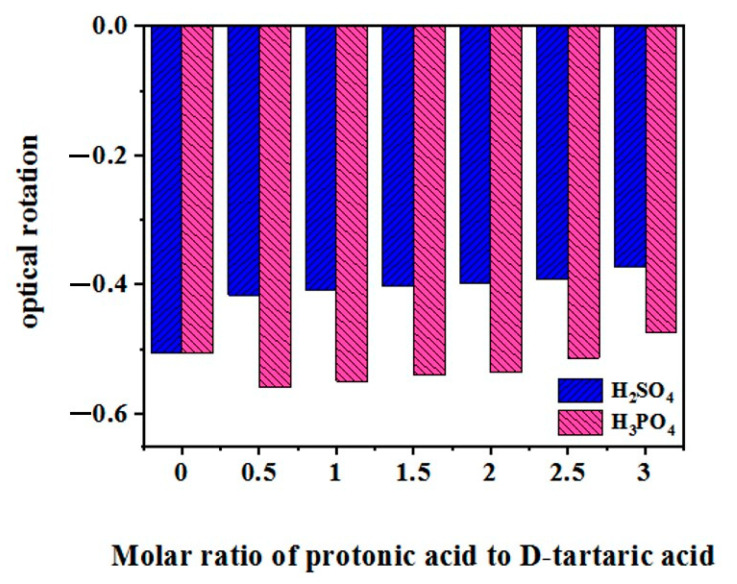
Effect of protonic acids on the rotation of D-(−)-tartaric acid.

The addition of protonic acids such as sulfuric acid and phosphoric acid also affected the optical rotation of the aqueous solution of a given HCA (Figure 4). As shown in Figure 4, the addition of sulfuric acid to the D-(−)-TA solution decreases the optical rotation, while the addition of phosphoric acid increases and then decreases the optical rotation. This phenomenon was possibly attributed to the attack of hydrogen ions on the hydroxyl groups under strongly acidic conditions, leading to partial inversion of the configuration of the chiral carbon. The increase in optical rotation with the addition of phosphoric acid was possibly due to the formation of a complex between the phosphoric acid and D-(−)-TA. With an increase in phosphoric acid concentration, the optical rotation decreased again due to the partial inversion of the configuration of the chiral carbon.

Unlike strong protonic acids such as sulfuric acid, the absolute value of the optical rotation of a chiral HCA solution gradually increased as BA formed complexes with chiral HCA, with increasing BA-to-HCA molar ratio. The effect of BA on the optical rotation of the aqueous solution of chiral HCA, as measured in terms of the maximum rate of change, decreases in the following order: L-TA (267%) ≈ D-(−)-TA (261%) > L-H_2_Mi (88%) > D-GlcA (66%) > R-MA (13%) > D-LA (−13%), as shown in Figure 2. The two enantiomers, L(+)-TA and D(−)-TA, demonstrated an increase in the absolute value of the optical rotation with increasing molar ratio of BA to tartaric acid, forming two curves symmetric to each other with respect to line y = 0. This indicated the two enantiomers of a given HCA reacted with BA in a similar stoichiometric manner. As shown above, the more hydroxyl and carboxyl groups are attached to the alkyl chain connected to the chiral carbon, the greater the rate of change of the optical rotation.

As shown in Figure 3, the rate of change of the optical rotation gradually decreases for the aqueous solution of gluconic acid as the BA-to-HCA molar ratio increases, whereas it initially increases and then decreases for the aqueous solution of lactic acid, and then gradually decreases and approaches zero for the aqueous solutions of tartaric acid, malic acid, and mandelic acid (Figure 3). Gluconic acid contained multiple hydroxyl groups, of which four (in addition to the carboxyl group) could complex with BA to increase the optical rotation. The optical rotation of the aqueous solution of gluconic acid continuously increased over the examined range of the molar ratio of BA to gluconic acid, while the rate of change of the optical ratio continued to drop without showing a minimum value.

Our previous work showed as catalysts for the hydration reaction of α-pinene, the synergistic catalytic abilities of BA, phosphoric acid, and sulfuric acid with HCA decreased sequentially [27,29]. This was possibly related to the fact that BA was more likely to form a complex with HCA, which could better promote the forward shift of the ionization equilibrium of HCA.

### 2.3. Complexes between BA and Several Common HCAs

Glycolic acid, lactic acid, mandelic acid, gluconic acid, malic acid, tartaric acid, and citric acid are selected as the seven common α-HCAs, whose possible complexes with BA are shown in the Supplementary Information (Figures S1–S11). As shown in Figures S1–S11, glycolic acid, lactic acid, and mandelic acid can only form five-membered ring complexes with BA. When a monoligand complex formed, no new chiral center was generated in the complex. However, when a double-ligand complex formed, a new chiral center was generated at the connecting atom of the two rings, i.e., the boron atom served as the new chiral center. Although a new chiral center was generated when glycolic acid formed a diligand complex with BA, equal amounts of levorotatory and dextrorotatory enantiomers were produced due to the random nature of the complexation, resulting in a racemic mixture.

As shown in Figures S4–S9, gluconic acid, malic acid, and tartaric acid can form both five-membered ring and six-membered ring complexes with BA. Similarly, when BA complexed with HCA at a 1:1 ratio, no new chiral center was generated in the complex. When forming a double-ligand complex, a new chiral center was generated, which served as the connecting atom of the two rings, i.e., the boron atom became the new chiral center. Both gluconic acid and tartaric acid contained more than four hydroxyl groups and contained more types of complexes with BA than malic acid. For simplicity, only the types involving a carboxylic acid group as the complexing group were discussed in this work.

As shown in Figure S10, when BA is bound with citric acid at a 1:1 ratio or 1:2 ratio to form five-membered ring complexes, only the double-ligand complexes have a chiral center, i.e., a new chiral center is generated at the boron atom. However, when citric acid formed six-membered ring complexes with BA, a new chiral center was generated in both the single-ligand and double-ligand complexes (Figure S11). We observed 1:1 complexation generated a chiral center at the α-carbon of citric acid, while 1:2 complexation generated two new chiral centers, namely, one at the boron atom and the other at the α-carbon of citric acid. When citric acid formed six-membered ring complexes with BA, a total of eight enantiomers were generated, two of which were single-ligand complexes and four were double-ligand complexes. When citric acid complexed with BA, complexation occurred in a random manner, leading to equal amounts of left and right enantiomers, i.e., a stalemate. This was also confirmed through optical rotation measurements, i.e., the addition of BA to aqueous citric acid solution with various molar ratios of BA to citric acid led to zero optical rotation.

Changes in the optical rotation could be better explained through the configuration analysis of the BA-HCA complexes. (1) When complexes formed, the optical rotation of the aqueous solution of a given HCA increased due to the formation of a ring structure and the increase in electron polarizability. (2) New chiral centers were generated as double-ligand complexes formed; however, levorotatory and dextrorotatory enantiomers were produced in equal amounts, leading to a zero net contribution of newly formed chiral centers with an increase in optical rotation. Specifically, the aqueous solution of an HCA with a zero optical rotation continued to show zero optical rotation, even after the addition of BA, while the optical rotation of the aqueous chiral HCA solution increased after the addition of BA, which was attributed to the formation of single-ligand complexes.

According to complex configuration analysis and the formation equations of the BA-HCA complexes, it was evident the more types of complexes formed after the addition of BA into the aqueous solution of an ionizable HCA, the more anions were consumed, and the easier it became to shift the ionization equilibrium in the forward direction [28]. From a thermodynamic perspective, the more types of complexes formed, the greater the increase in entropy, and the easier it was for complexation to occur. Gluconic acid, for example, was a monocarboxylic acid containing multiple hydroxyl groups. Its solution pH decreased after the addition of BA, and it decreased significantly more than after the addition of glycolic acid, lactic acid, and mandelic acid as the molar ratio of BA to gluconic acid increased.

### 2.4. Boric Acid Catalyzed the Esterification of HCA

In previous studies, we found the composite catalyst of BA and tartaric acid could effectively catalyze the reaction of palmitic acid with methanol, with a yield of 98% for methyl palmitate [28]. Due to the difficulty of dissolving tartaric acid in palmitic acid, we speculated under the catalysis of BA, tartaric acid first reacted with methanol to form methyl tartrate, which then underwent an ester exchange reaction with palmitic acid to form fatty acid methyl esters. Nigiz et al. [30] used carboxymethyl cellulose membranes coated with BA to catalyze the esterification of lactic acid with ethanol at a reaction temperature of 75 °C. BA heated to 100–105 °C would lose one molecule of water and form metaboric acid, while BA subjected to prolonged heating in the temperature range of 104–160 °C could produce pyroboric acid. However, the stability of boronic acid complexes formed with HCAs at high temperatures (>100 °C) has been seldom reported in the literature.

In this study, the stability of the BA–HCA complexes at high temperatures (>100 °C) was examined through the catalytic synthesis of high-molecular-weight α-hydroxycarboxylic esters (TBC, TOC). BA was an efficient catalyst, showing catalytic ability at a molar ratio as low as 1‰ relative to the HCAs. For example, the 8 h yield of TBC was 87.8% when the molar ratio of BA to citric acid was 0.5‰, while the molar ratio of butanol to citric acid was 5:1, and the reaction temperature was 100–110 °C. The yield of TBC, however, was zero in the absence of the catalyst, while the experimental conditions remained unchanged. As the molecular weight of the alcohol increased, the temperature required for esterification also increased. For example, the yield of TOC after 8 h was 93.8% when the molar ratio of citric acid to octanol was 1:5, the dosage of BA was 0.5% as a mass ratio relative to citric acid, and the reaction temperature was 110–170 °C. Moreover, B_2_O_3_ could be used as a catalyst instead of BA, which indicated the complexes were still active at 100–170 °C.

At the end of the esterification reaction, there was no need to separate the catalyst if acetylation of the hydroxyl groups of the HCAs was required. For the acetylation process to occur, the unreacted alcohol only had to be removed by distillation, followed by the addition of acetic anhydride at 80–100 °C. After the BA-catalyzed synthesis of TBC and TOC, the acetylation of ATBC and ATOC could be carried out without separating and replacing the catalyst, which was very beneficial for industrial production. After acetylation, the products could be neutralized with a sodium carbonate solution and the washing solution was yellow in color.

The refractive indices of products TBC, ATBC, TOC, and ATOC at room temperature were measured as 1.4433° (25.7 °C), 1.4420° (26.1 °C), 1.4533° (26.1 °C), and 1.4528° (26.1 °C), respectively, which was in agreement with the values reported in the literature [31,32]. The IR, ^1^H-NMR, and ^13^C-NMR spectra of the TBC, ATBC, TOC, and ATOC products are shown in the Supplementary Information (Figures S12–S14), where spectral interpretation is performed according to references [31,32,33].

**Figure 5:**
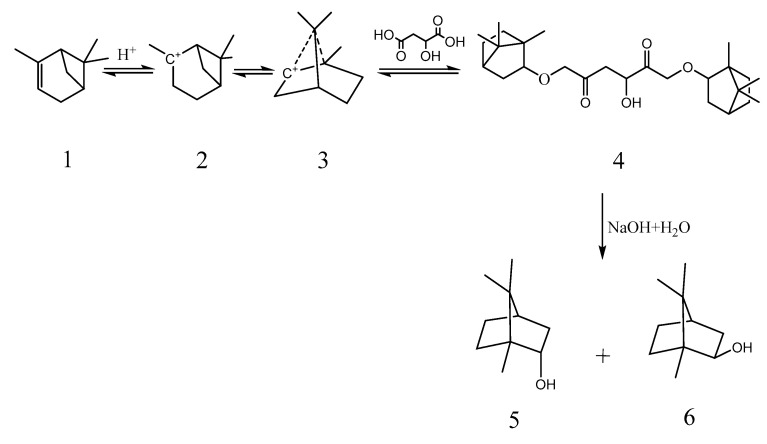
The reaction process of synthetic borneol. 1. α-pinene; 2. carbocation; 3. carbocation; 4. bornyl malate; 5. borneol; 6. Isobornyl.

**Figure 6:**
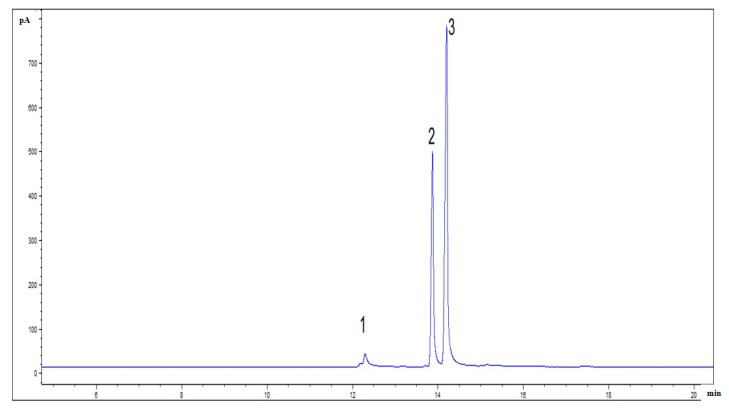
Gas chromatography (GC) spectrum of crude borneol after the saponification reaction. Note: 1. fenicol, 2. isoborneol, 3. borneol.

Interestingly, BA could also catalyze the esterification reaction between malic acid and pinene. After the reaction was complete, a saponification reaction was carried out to obtain borneol with optical activity. The mass ratio of (−)-α-pinene, malic acid, and BA was 13.6:(6–13.4):(0.15–0.6), the reaction temperature was 50 °C, and the reaction time was 20 h. The product was saponified with a 30% sodium hydroxide solution and then distilled with steam to produce borneol. After saponification, the product yield was 92–95% (pinene mass), and the GC content of borneol was 40–45%. The product was subjected to vacuum fractionation, and the GC content of the borneol was 96.5%, where the mass yield of crud borneol was 39–42% (pinene mass). The reaction process of synthetic borneol is shown in Figure 5. Figure 6 presents the gas chromatography (GC) plot of crude borneol, with a content of 34.6% for isoborneol and 61.9% for borneol. After dissolving the synthesized borneol in ethanol, the specific rotation was measured as −25.1° at 20 °C. The optical rotation direction of the product borneol was consistent with that of raw material pinene, where left-handed pinene could yield left-handed borneol, while right-handed pinene could yield right-handed borneol. If boron oxide was used instead of BA in this reaction, it would lead to an increase in the formation of polymerization products and a blackening of the color.

Boric acid was used to catalyze the esterification of α-pinene with malic acid. At the same temperature and reaction time, when malic acid was used as a catalyst alone, the conversion rate of pinene was approximately 1%, and the product was camphene. When boric acid was used as a catalyst alone, the conversion rate of pinene was almost zero. Therefore, we inferred the catalyst was a complex composed of boric acid and malic acid, which was also supported by pH and optical rotation measurements [28]. The complexation reaction between HCAS such as malic acid and boric acid was dynamic and reversible. Therefore, the acidity of malic acid increased; however, a C-O-B bond formed between the carboxyl and hydroxyl groups of malic acid and the hydroxyl group of boric acid when the complex formed. Therefore, the reduction in polarity made it more soluble in non-polar pinene, which was more conducive to the esterification reaction.

The synthesized borneol was recrystallized in anhydrous ethanol to obtain a borneol sample with a GC content of 99%. The hydrogen and carbon nuclear magnetic resonance spectra of synthetic borneol are shown in Figures S15 and S16 in the Supplementary Information, respectively. The NMR results were as follows: ^1^H NMR (300 MHz, CDCl_3_) *δ* 5.38 (d, *J* = 3.0 Hz, 1H), 2.09–1.97 (m, 4H), 1.65 (s, 3H), 1.57–1.45 (m, 3H), 1.18 (d, *J* = 3.0 Hz, 6H); ^1^C NMR (75 MHz, CDCl_3_) *δ* 134.05, 120.54, 44.99, 31.01, 27.44, 26.89, 26.25, 23.97, 23.37, and 23.12.

From these results, we inferred the complex composed of BA and HCA played a catalytic role, rather than BA. The catalyst composed of BA and HCA was likely the first form the intermediates of hydroxycarboxylic acid esters in the reaction, such as methyl tartrate and terpinyl tartrate. Then, methyl tartrate underwent ester exchange with palmitic acid to generate methyl palmitate, and turpentyl tartrate was hydrolyzed to obtain terpineol. Because the intermediate hydroxycarboxylic acid methyl ester was more easily generated than hydroxy acid terpinyl ester and borneol ester, when BA and HCA were used as catalysts, the yield of palmitic acid methyl ester could reach 98%, the yield of terpineol was about 55%, and the yield of borneol was less than 40% [27,28].

## 3. Experimental Section

### 3.1. Materials and Apparatus

The following starting materials and reagents were used in this study: glycolic acid (98%), citric acid (99.5%), L-TA (99.5%), D-(−)-tartaric acid (99.5%), D-LA (90%), R-MA (99%), L-H_2_Mi, D-GlcA (49–53wt.% in H_2_O), boron oxide (98%), and (−)-α-pinene (98%). These compounds were purchased from Shanghai McLin Biochemical Technology (Shanghai, China). Sulphuric acid (98%), phosphoric acid (85%), methanol (99.5%), n-butanol (99.5%), sec-butanol (99%), 1-octanol (99%), 2-ethylhexanol (99%), sodium hydroxide (98%), and sodium carbonate anhydrous (98%) were purchased from Chengdu Kelong Chemical (Chengdu, Sichuan Province, China). All reagents were analytically pure. Distilled water was produced in the laboratory.

The reaction apparatus consisted of an organic synthesis unit PPV-3000 (EYELA, Tokyo Rikakikai, Tokyo, Japan). The following analytical instruments were used in this work: an SGW-2 automatic polarimeter (Shanghai Jingke, Shanghai, Chinese) with a test tube length of 100 mm, and a light source wavelength of 589 nm. In addition, we used a WAY-2W Abbe refractometer (Shanghai Optical Instrument Factory, Shanghai, China), a near infrared quality analyzer, SY-3650-II, Foss NIRSystems Inc., an AVANCE III 300M or 600M nuclear magnetic resonance spectrometer (Bruker, Switzerland), a 7890A gas chromatograph (Agilent, Santa Clara, CA, USA) equipped with quartz capillary chromatography columns (60 m × 0.25 mm × 0.25 µm) with AT-35 as the immobile phase, and a TQ456 GC-MS instrument (Bruker, Billerica, MA, USA) equipped with BR-5 elastic quartz capillary columns (30 m × 0.25 mm × 0.25 m) as the chromatography columns.

### 3.2. Experimental Methods

#### 3.2.1. Determination of the Optical Rotation of the BA and HCA Mixture

The HCAs were prepared with a concentration of 0.2 mol/kg using distilled water. Then, BA was added at different proportions into three replicates of the HCA solutions, and the optical rotations of the three replicate solutions were measured. The ratio of change in the optical rotation of the HCAs was calculated by

(α_0_ − α_1_)/α_0,_

where α_0_ is the optical rotation of the HCAs at the given concentration (i.e., 0.2 mol/kg) and α_1_ is the optical rotation after BA addition.

#### 3.2.2. α-Hydroxycarboxylic Esters

In a three-necked flask, 0.2 mol of citric acid, 1.0 mol of butanol/octanol, and 0.001 mol of BA were added and combined with toluene as the water-carrying agent. The reaction mixture was mechanically stirred at 400 rpm, and the reaction temperature was controlled at 95–160 °C. The reaction-generated water was separated through an oil-water separator, and the reaction was stopped when no more water emerged from the oil-water separator. The toluene and unreacted alcohol were evaporated under reduced pressure, and the product was poured into a separatory funnel, where it was neutralized with a sodium carbonate solution, washed with water, and dried before sampling and analysis.

To synthesize chiral borneol, 13.6 g of (−)-α-pinene, 4–13.4 g of malic acid, and 0.06–0.6 g of BA were added to a reaction flask at a temperature of 50–60 °C and a reaction time of 3–5 h. After the reaction was complete, 60 g of 30% sodium hydroxide solution was added for a saponification reaction, and borneol was evaporated with water vapor.

### 3.3. Analytical Methods

Infrared spectral data acquisition and mass spectrometry were conducted according to the methods described in reference [28]. For H NMR data acquisition, GC analysis, and GC-MS analysis were conducted according to the methods described in reference [34].

## 4. Conclusions

The mathematical expressions for optical rotation as functions of the BA-to-HCA molar ratio were obtained through polynomial fitting. Functions linking the rate of change of the optical rotation and BA-to-HCA molar ratio were obtained by differentiating the mathematical expressions. The results showed the rate of change of the optical rotation gradually decreased as the BA-to-HCA molar ratio increased, reaching a minimum value at a molar ratio of approximately three.

Due to the promoting effect of BA on the deprotonation of HCAs, BA also served as an excellent catalyst for the esterification of HCAs, demonstrating excellent catalytic performance at a mass ratio of as low as 1‰ relative to the HCAs. As a catalyst, BA could catalyze the acetylation of the hydroxyl groups in addition to the esterification of HCAs. Compared to the conventional synthesis routes of ATBC and ATOC, a synthesis route with BA as the catalyst allowed for a lower frequency of catalyst separation and replacement while providing light-colored products.

Borneol was synthesized by catalyzing the reaction of malic acid and α-pinene with BA and then performing a saponification reaction. The reaction conditions of this method were relatively mild, and the obtained borneol product exhibited optical activity. This solved the disadvantages of the traditional oxalic acid method, which has shown difficulty controlling the reaction condition, rapid explosion, low yield, and high content of isobornol in the product [35,36]. The amount of boric acid catalyst used in this reaction was small, and the catalyst used in the reaction consisted of a strong acidic complex formed by boric acid and malic acid, that was, the catalyst consisted of the formed complex.

## Data Availability

Data are contained within the article and Supplementary Materials.

## Supplementary Materials

The following supporting information can be downloaded at: https://www.mdpi.com/article/10.3390/molecules29010043/s1.
