# Supplementary material for: Complexation of Boronic Acid with Chiral α-Hydroxycarboxylic Acids and the Ability of the Complexes to Catalyze α-Hydroxycarboxylic Acid Esterification
Source: Molecules. 2023 Dec 20;29(1):43. doi: 10.3390/molecules29010043 (PMC10779702; doi:10.3390/molecules29010043)
Supplement: Supplementary file 1 [file molecules-29-00043-s001.zip › molecules-2758266-supplementary.pdf]

Supplementary Information

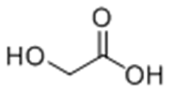

glycolic acid

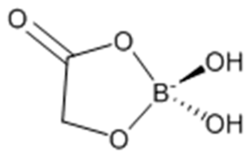

2,2-dihydroxy-4-oxo-1,3,2-dioxaborolan-2-uide

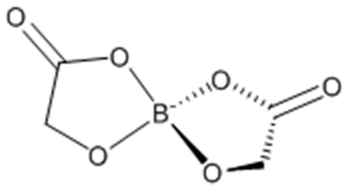

(*S*)-2,7-dioxo-1,4,6,9-tetraoxa-5-boraspiro[4.4]nonan-5-uide

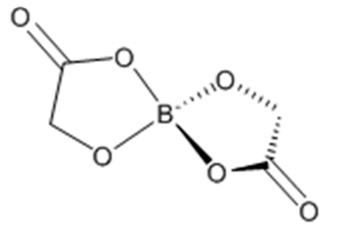

(*R*)-2,7-dioxo-1,4,6,9-tetraoxa-5-boraspiro[4.4]nonan-5-uide

Figure S1. Complexes formed by glycolic acid and boric acid.

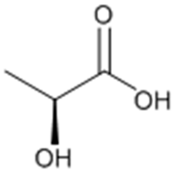

(*S*)-2-hydroxypropanoic acid

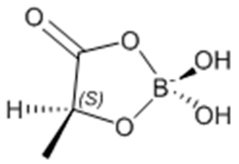

(*S*)-2,2-dihydroxy-4-methyl-5-oxo-1,3,2-dioxaborolan-2-uide

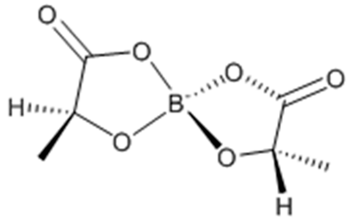

(2*S*,5*S*,7*S*)-2,7-dimethyl-3,8-dioxo-1,4,6,9-tetraoxa-5-boraspiro[4.4]nonan-5-uide

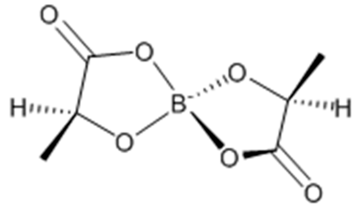

(2*S*,5*R*,7*S*)-2,7-dimethyl-3,8-dioxo-1,4,6,9-tetraoxa-5-boraspiro[4.4]nonan-5-uide

Figure S2. Complexes formed by D-(-)-lactic acid and boric acid.

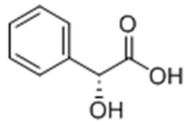

(*R*)-2-hydroxy-2-phenylacetic acid

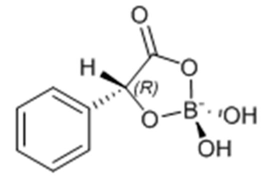

(*R*)-2,2-dihydroxy-4-oxo-5-phenyl-1,3,2-dioxaborolan-2-uide

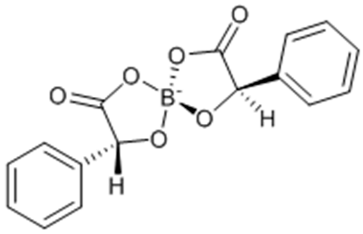

(3*R*,5*S*,8*R*)-2,7-dioxo-3,8-diphenyl-1,4,6,9-tetraoxa-5-boraspiro[4.4]nonan-5-uide

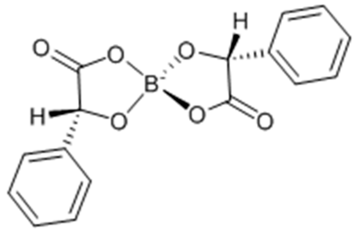

(3*R*,5*R*,8*R*)-2,7-dioxo-3,8-diphenyl-1,4,6,9-tetraoxa-5-boraspiro[4.4]nonan-5-uide

Figure S3. Complexes formed by (R)-(-)-mandelic acid and boric acid.

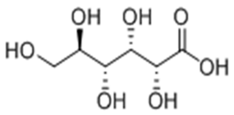

(2*R*,3*S*,4*R*,5*R*)-2,3,4,5,6-pentahydroxyhexanoic acid

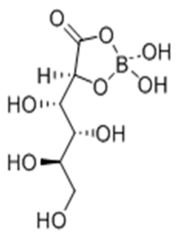

(*S*)-2,2-dihydroxy-4-oxo-5-((1*S*,2*R*,3*R*)-1,2,3,4-tetrahydroxybutyl)-1,3,2-dioxaborolan-2-uide

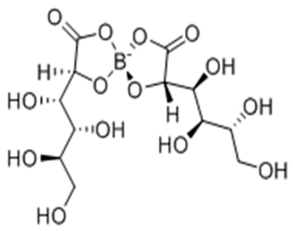

(3*S*,5*S*,8*S*)-2,7-dioxo-3,8-bis((1*S*,2*R*,3*R*)-1,2,3,4-tetrahydroxybutyl)-1,4,6,9-tetraoxa-5-boraspiro[4.4]nonan-5-uide

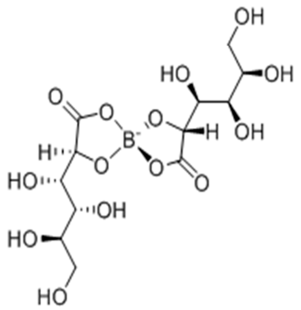

(3*R*,5*R*,8*S*)-2,7-dioxo-3,8-bis((1*S*,2*R*,3*R*)-1,2,3,4-tetrahydroxybutyl)-1,4,6,9-tetraoxa-5-boraspiro[4.4]nonan-5-uide

Figure S4. Complexes of D-gluconic acid with boric acid forming five-membered rings.

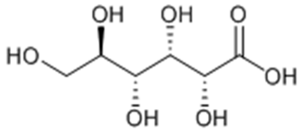

(2*R*,3*S*,4*R*,5*R*)-2,3,4,5,6-pentahydroxyhexanoic acid

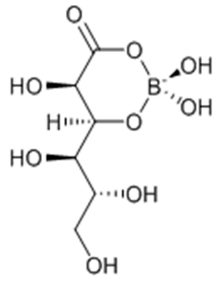

(5*R*,6*S*)-2,2,5-trihydroxy-4-oxo-6-((1*R*,2*R*)-1,2,3-trihydroxypropyl)-1,3,2-dioxaborinan-2-uide

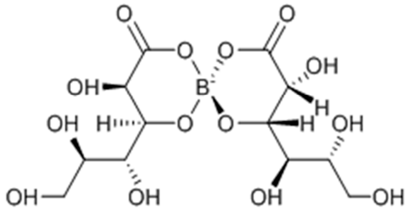

(3*R*,4*S*,6*S*,9*R*,10*S*)-3,9-dihydroxy-2,8-dioxo-4,10-bis((1*R*,2*R*)-1,2,3-trihydroxypropyl)-1,5,7,11-tetraoxa-6-boraspiro[5.5]undecan-6-uide

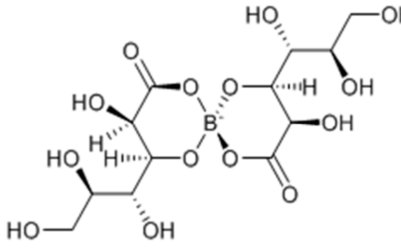

(3*R*,4*S*,6*R*,9*R*,10*S*)-3,9-dihydroxy-2,8-dioxo-4,10-bis((1*R*,2*R*)-1,2,3-trihydroxypropyl)-1,5,7,11-tetraoxa-6-boraspiro[5.5]undecan-6-uide

Figure S5. Complexes of D-gluconic acid with boric acid forming six-membered rings.

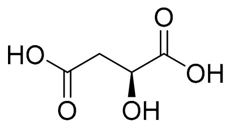

(*S*)-2-hydroxysuccinic acid

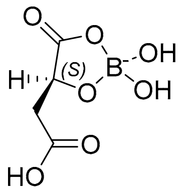

(*S*)-4-(carboxymethyl)-2,2-dihydroxy-5-oxo-1,3,2-dioxaborolan-2-uide

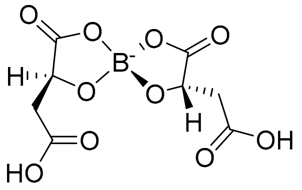

(2*S*,5*S*,7*S*)-2,7-bis(carboxymethyl)-3,8-dioxo-1,4,6,9-tetraoxa-5-boraspiro[4.4]nonan-5-uide

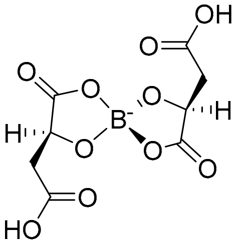

(2*S*,5*R*,7*S*)-2,7-bis(carboxymethyl)-3,8-dioxo-1,4,6,9-tetraoxa-5-boraspiro[4.4]nonan-5-uide

Figure S6. Complexes of L-(-)-malic acid with boric acid forming five-membered rings.

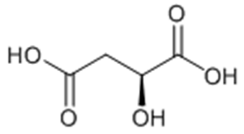

(*S*)-2-hydroxysuccinic acid

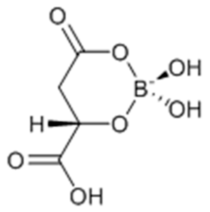

(*S*)-4-carboxy-2,2-dihydroxy-6-oxo-1,3,2-dioxaborinan-2-uide

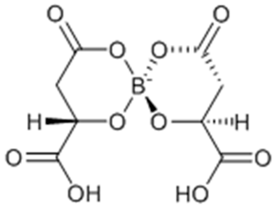

(2*S*,6*S*,8*S*)-2,8-dicarboxy-4,10-dioxo-1,5,7,11-tetraoxa-6-boraspiro[5.5]undecan-6-uide

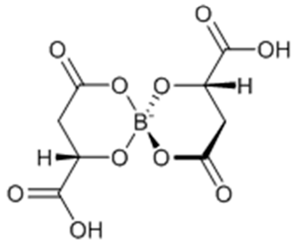

(2*S*,6*R*,8*S*)-2,8-dicarboxy-4,10-dioxo-1,5,7,11-tetraoxa-6-boraspiro[5.5]undecan-6-uide

Figure S7. Complexes of L-(-)-malic acid with boric acid forming six-membered rings.

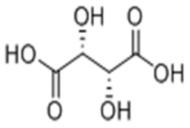

(2*R*,3*R*)-2,3-dihydroxysuccinic acid

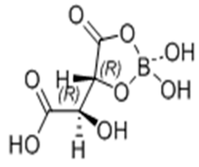

(*R*)-4-((*R*)-carboxy(hydroxy)methyl)-2,2-dihydroxy-5-oxo-1,3,2-dioxaborolan-2-uide

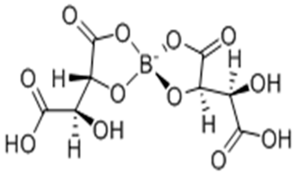

(2*R*,5*S*,7*R*)-2,7-bis((*R*)-carboxy(hydroxy)methyl)-3,8-dioxo-1,4,6,9-tetraoxa-5-boraspiro[4.4]nonan-5-uide

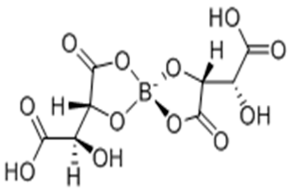

(2*R*,5*R*,7*R*)-2,7-bis((*R*)-carboxy(hydroxy)methyl)-3,8-dioxo-1,4,6,9-tetraoxa-5-boraspiro[4.4]nonan-5-uide

Figure S8. Complexes of L-(+)-tartaric acid with boric acid forming five-membered rings.

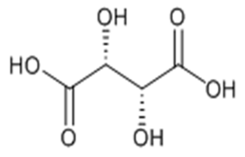

(2*R*,3*R*)-2,3-dihydroxysuccinic acid

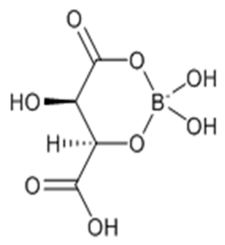

(4*R*,5*R*)-4-carboxy-2,2,5-trihydroxy-6-oxo-1,3,2-dioxaborinan-2-uide

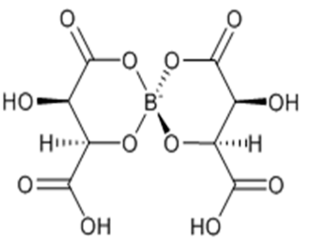

(2*R*,3*R*,6*S*,8*S*,9*S*)-2,8-dicarboxy-3,9-dihydroxy-4,10-dioxo-1,5,7,11-tetraoxa-6-boraspiro[5.5]undecan-6-uide

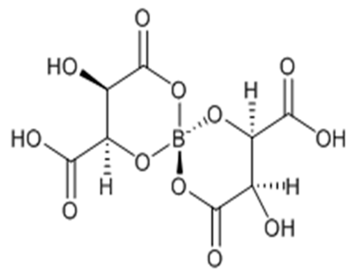

(2*R*,3*R*,6*R*,8*R*,9*R*)-2,8-dicarboxy-3,9-dihydroxy-4,10-dioxo-1,5,7,11-tetraoxa-6-boraspiro[5.5]undecan-6-uide

Figure S9. Complexes of L-(+)-tartaric acid with boric acid forming six-membered rings.

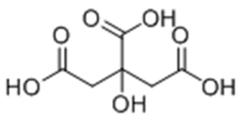

2-hydroxypropane-1,2,3-tricarboxylic acid

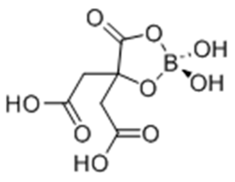

4,4-bis(carboxymethyl)-2,2-dihydroxy-5-oxo-1,3,2-dioxaborolan-2-uide

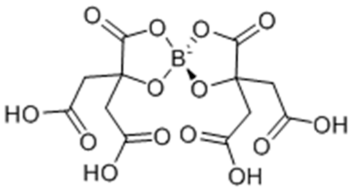

(*S*)-2,2,7,7-tetrakis(carboxymethyl)-3,8-dioxo-1,4,6,9-tetraoxa-5-boraspiro[4.4]nonan-5-uide

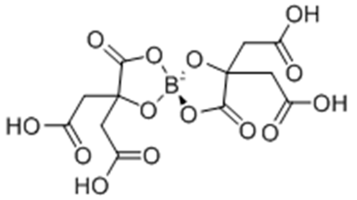

(*R*)-2,2,7,7-tetrakis(carboxymethyl)-3,8-dioxo-1,4,6,9-tetraoxa-5-boraspiro[4.4]nonan-5-uide

Figure S10. Complexes of citric acid with boric acid forming five-membered rings.

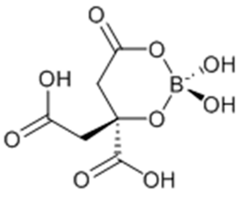

(*S*)-4-carboxy-4-(carboxymethyl)-
2,2-dihydroxy-6-oxo-1,3,2-dioxaborinan-2-uide

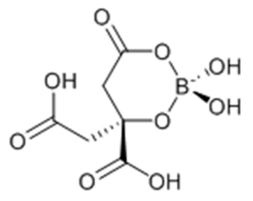

(*R*)-4-carboxy-4-(carboxymethyl)-
2,2-dihydroxy-6-oxo-1,3,2-dioxaborinan-2-uide

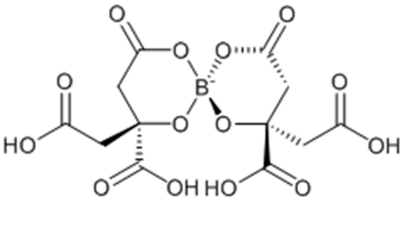

(2*S*,6*S*,8*S*)-2,8-dicarboxy-2,8-
bis(carboxymethyl)-4,10-dioxo-1,5,7,11-
tetraoxa-6-boraspiro[5.5]undecan-6-uide

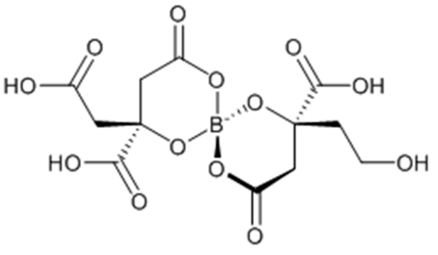

(2*S*,6*R*,8*S*)-2,8-dicarboxy-2-
(carboxymethyl)-8-(2-hydroxyethyl)-4,10-dioxo-
1,5,7,11-tetraoxa-6-boraspiro[5.5]undecan-6-uide

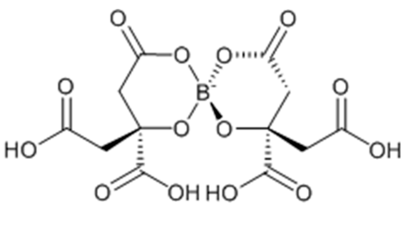

(2*R*,6*S*,8*S*)-2,8-dicarboxy-2,8-
bis(carboxymethyl)-4,10-dioxo-1,5,7,11-
tetraoxa-6-boraspiro[5.5]undecan-6-uide

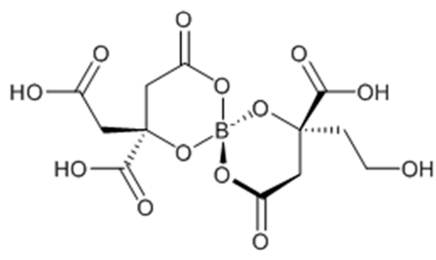

(2*S*,6*R*,8*R*)-2,8-dicarboxy-2-
(carboxymethyl)-8-(2-hydroxyethyl)-4,10-dioxo-
1,5,7,11-tetraoxa-6-boraspiro[5.5]undecan-6-uide

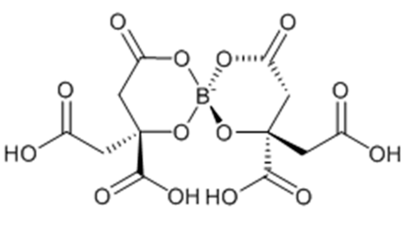

(2*R*,6*S*,8*R*)-2,8-dicarboxy-2,8-
bis(carboxymethyl)-4,10-dioxo-1,5,7,11-
tetraoxa-6-boraspiro[5.5]undecan-6-uide

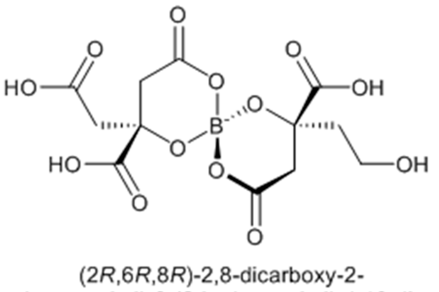

(2*R*,6*R*,8*R*)-2,8-dicarboxy-2-
(carboxymethyl)-8-(2-hydroxyethyl)-4,10-dioxo-
1,5,7,11-tetraoxa-6-boraspiro[5.5]undecan-6-uide

Figure S11. Complexes of citric acid with boric acid forming six-membered rings.

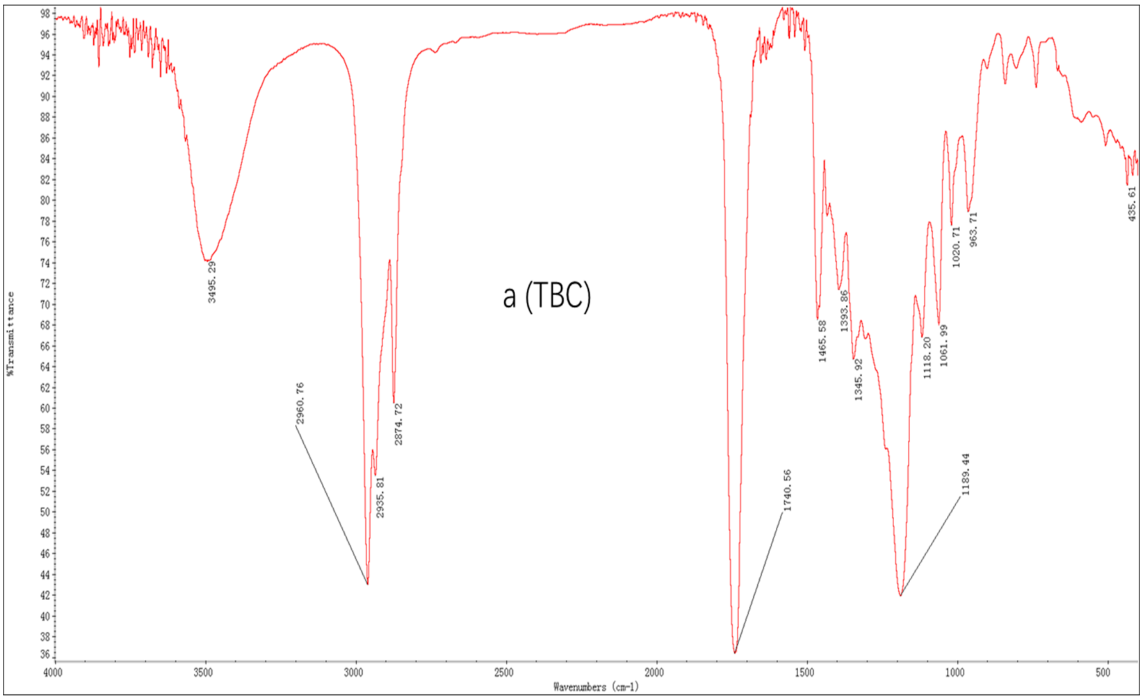
a (TBC)
%Transmittance
Wavenumbers (cm-1)
3495.29
2960.76
2935.81
2874.72
1740.56
1465.58
1393.86
1345.92
1189.44
1118.20
1061.99
1020.71
963.71
435.61

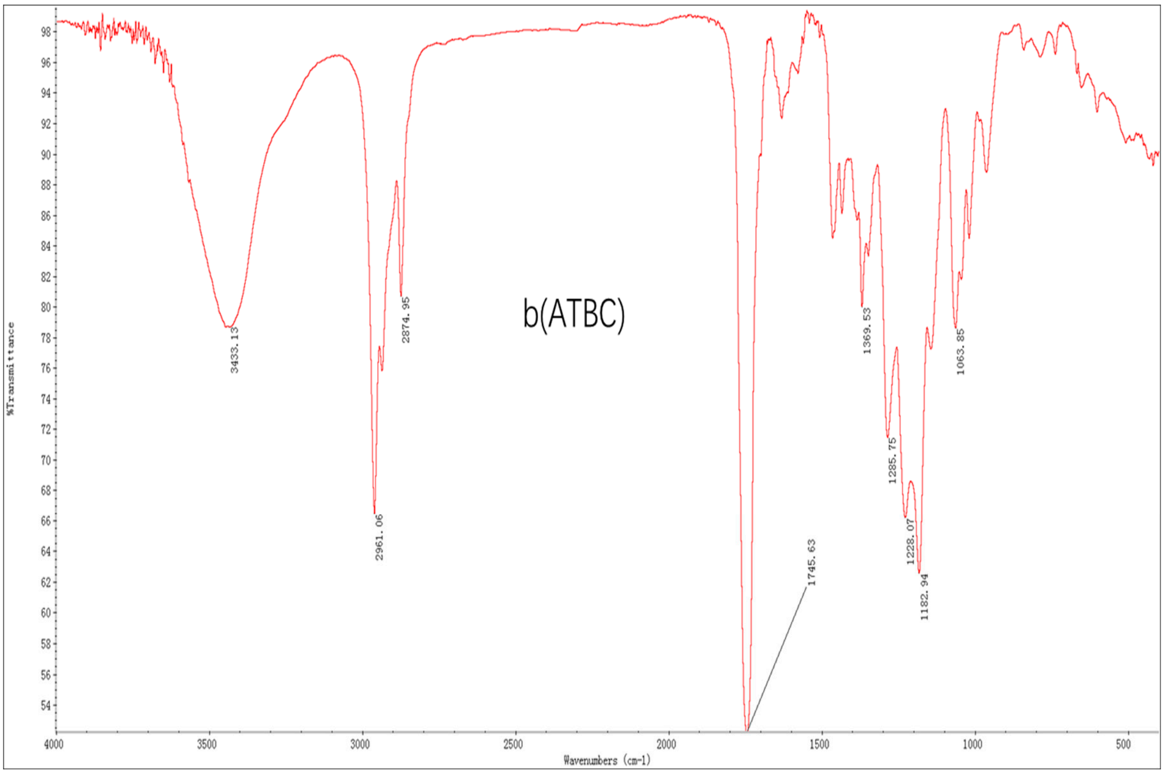
b(ATBC)
%Transmittance
Wavenumbers (cm-1)
3433.13
2961.06
2874.95
1745.63
1369.53
1285.75
1228.07
1182.94
1063.85

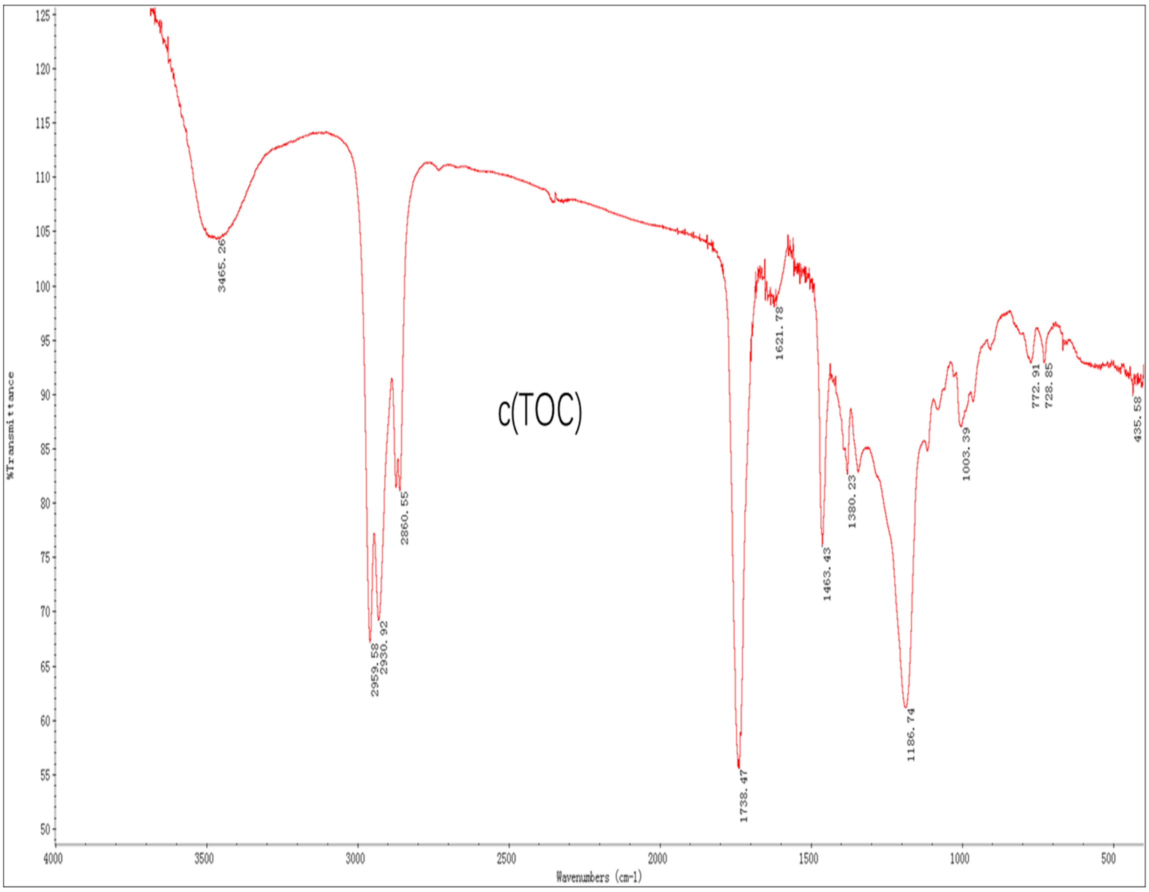

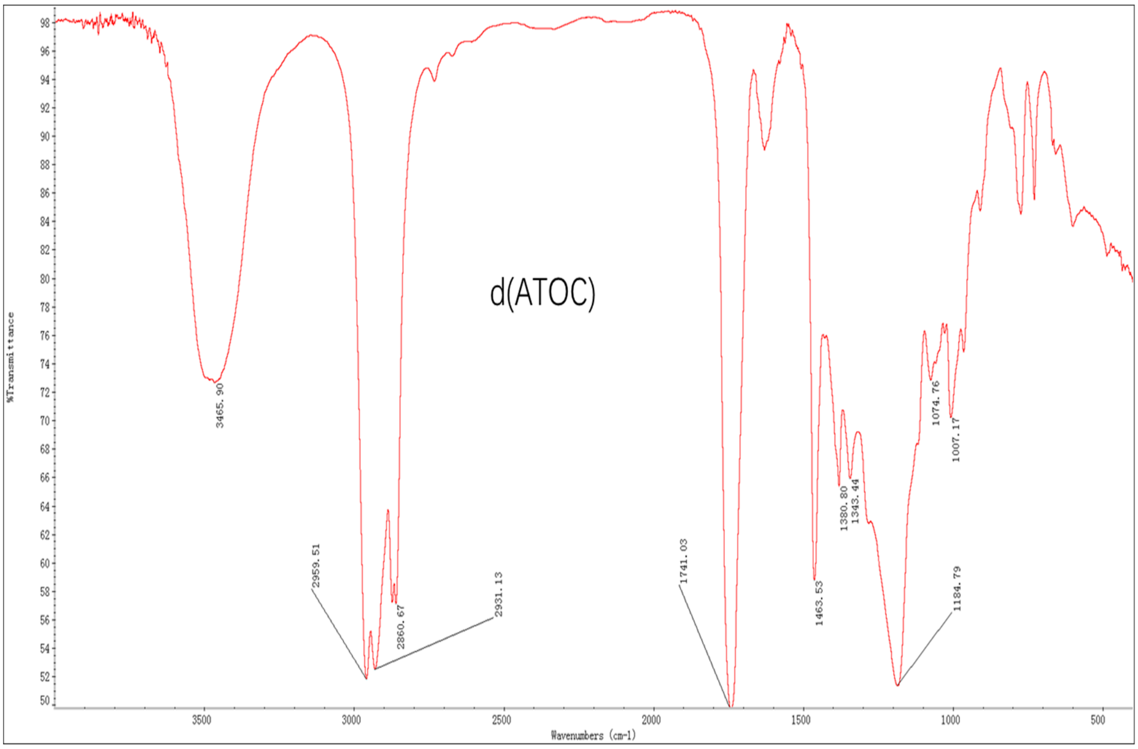

Figure S12. IR spectra of products TBC, ATBC, TOC, and ATOC. (a). The IR characteristic peaks appeared at the following wavenumbers (cm^{-1}): 3495 (-OH), 1740 (-C=O), 1189 (C-O-C), and 1062 (C-O).(b). The IR characteristic peaks appeared at the following wavenumbers (cm^{-1}): 1745 (-C=O), 1182 (C-O-C), and 1063 (C-O). (c). The IR characteristic peaks appeared at the following wavenumbers (cm^{-1}): 3522 (-OH), 1739 (-C=O), and 1188 (C-O-C). (d). The IR characteristic peaks appeared at the following wavenumbers (cm−1): 1741 (-C=O), 1184 (C-O-C), and 1074 (C-O).

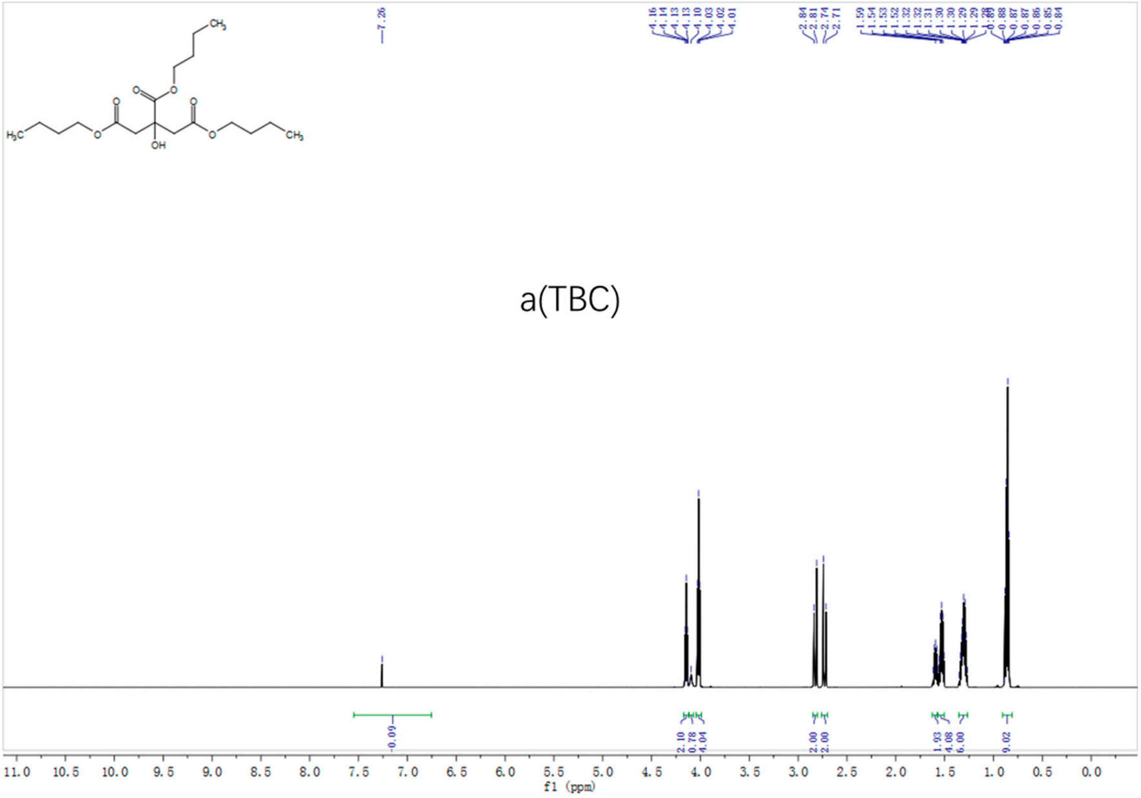
a(TBC)
f1 (ppm)

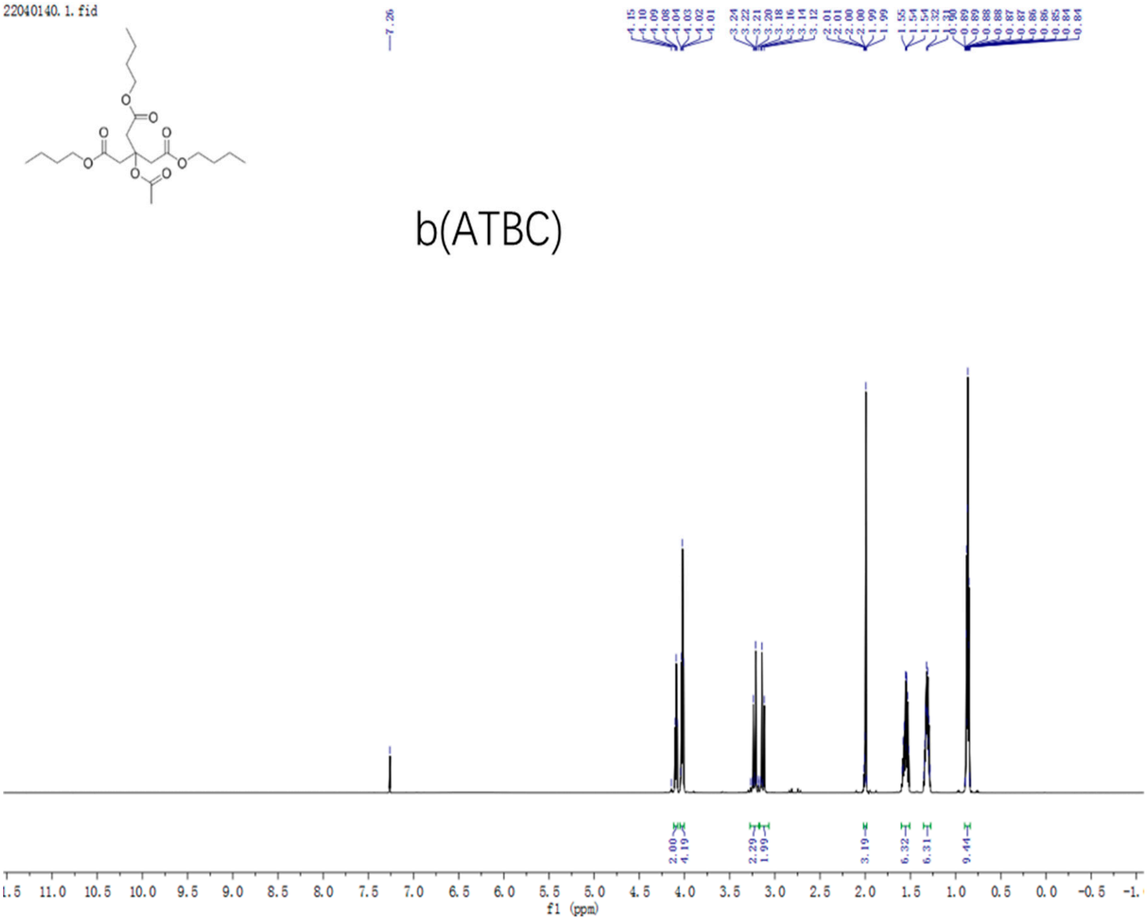
22040140.1.fid
b(ATBC)
f1 (ppm)

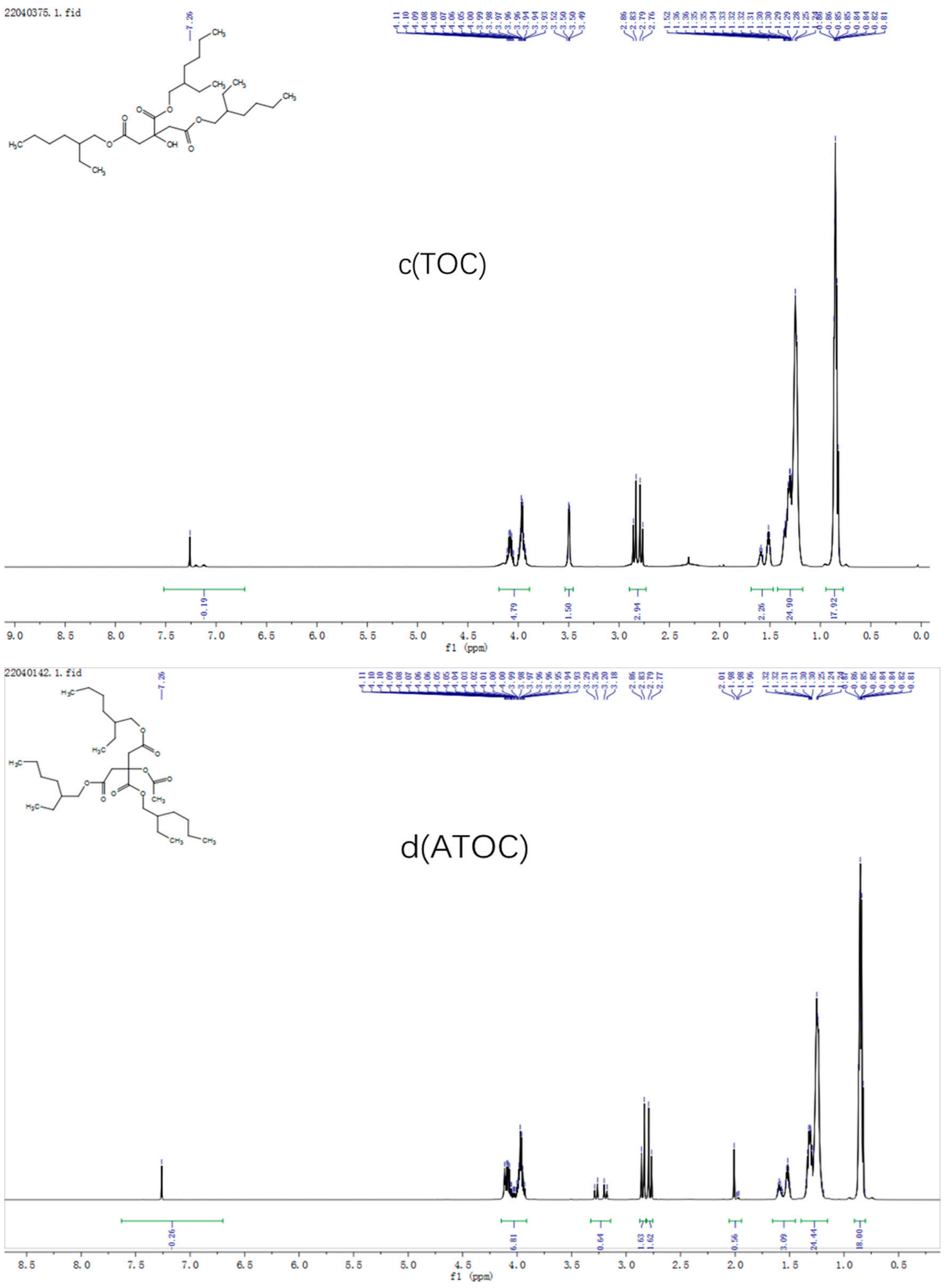

Figure S13. ^{1}H-NMR spectra of products TBC, ATBC, TOC, and ATOC. (a). The 1H NMR (600 MHz, $CDCl_3$) values were δ 4.14 (t, J =9.0 Hz, 2H), 4.10 (s, 1H), 4.02 (t, J = 6.0 Hz, 4H), 2.83 (d, J = 18 Hz, 2H), 2.73 (d, J = 18 Hz, 2H), 1.62–1.58 (m, 2H), 1.57–1.51 (m, 4H), 1.35–1.27 (m, 6H), 0.89–0.84 (m, 9H). (b). The 1H NMR (600 MHz, $CDCl_3$) values were δ 4.14 (t, J =9.0 Hz, 2H), 4.02 (t, J = 6.0 Hz, 4H), 2.83 (d, J = 18 Hz, 2H), 2.73 (d, J = 18 Hz, 2H), 2.00 (m, 3H), 1.57–1.51 (m, 6H), 1.35–1.27 (m, 6H), and 0.89–0.84 (m, 9H). (C). The 1H NMR (600 MHz, $CDCl_3$) values were δ 4.12–4.05 (m, 2H), 4.00–3.93 (m, 4H), 2.88–2.76 (m, 4H), 1.61–1.57 (m, 1H), 1.53–1.49 (m, 2H), 1.35–1.19 (m, 24H), and 0.88–0.80 (m, 18H). (d). The 1H NMR (600 MHz, $CDCl_3$) values were δ 4.15–3.93 (m, 7H), 3.29–3.18 (m, 1H), 2.85 (d, J = 12.0 Hz, 2H), 2.78 (d, J = 12.0 Hz, 2H), 2.01–1.96 (m, 1H), 1.61–1.50 (m, 3H), 1.32–1.24 (m, 24H), and 0.85–0.81 (m, 18H).

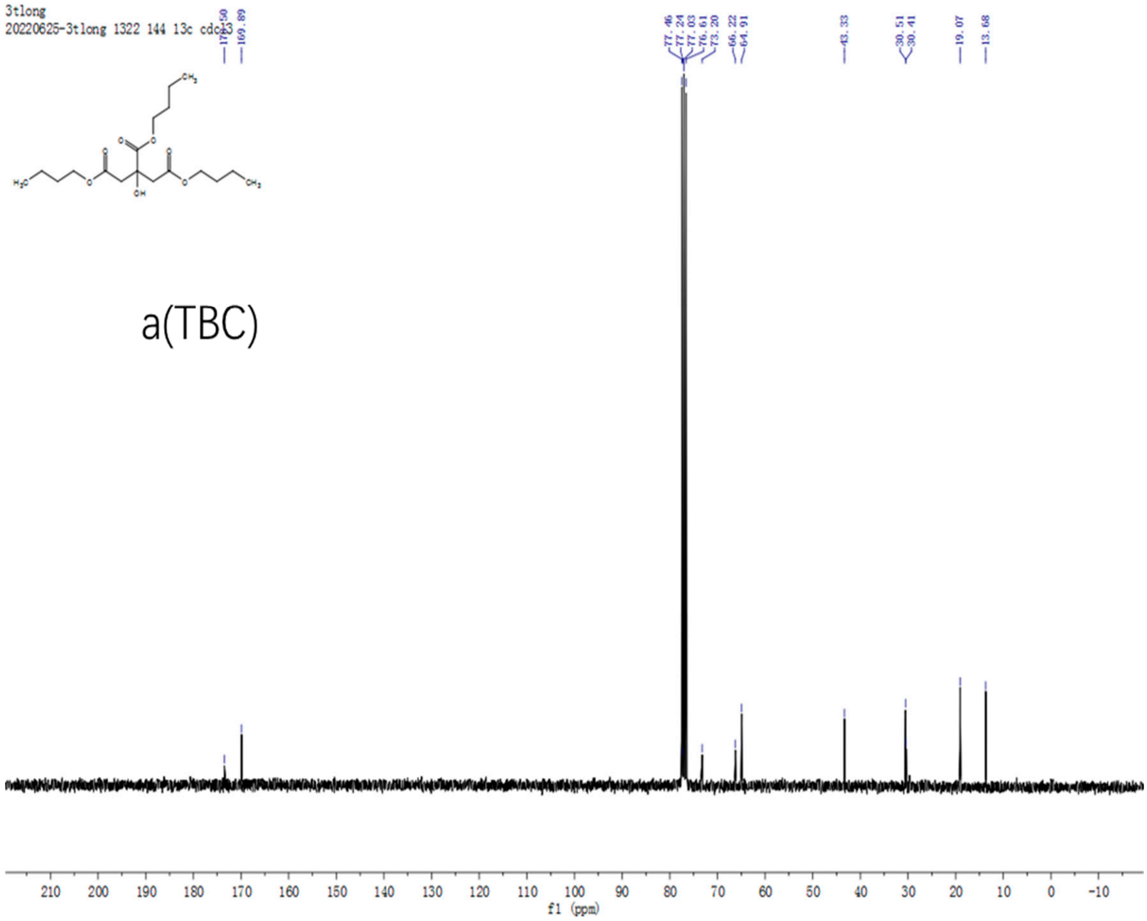
3tlong
20220625-3tlong 1322 144 13c cdcl3
169.89
77.46
77.24
77.03
76.61
73.20
66.22
64.91
43.33
30.51
30.41
19.07
13.68
a(TBC)
f1 (ppm)

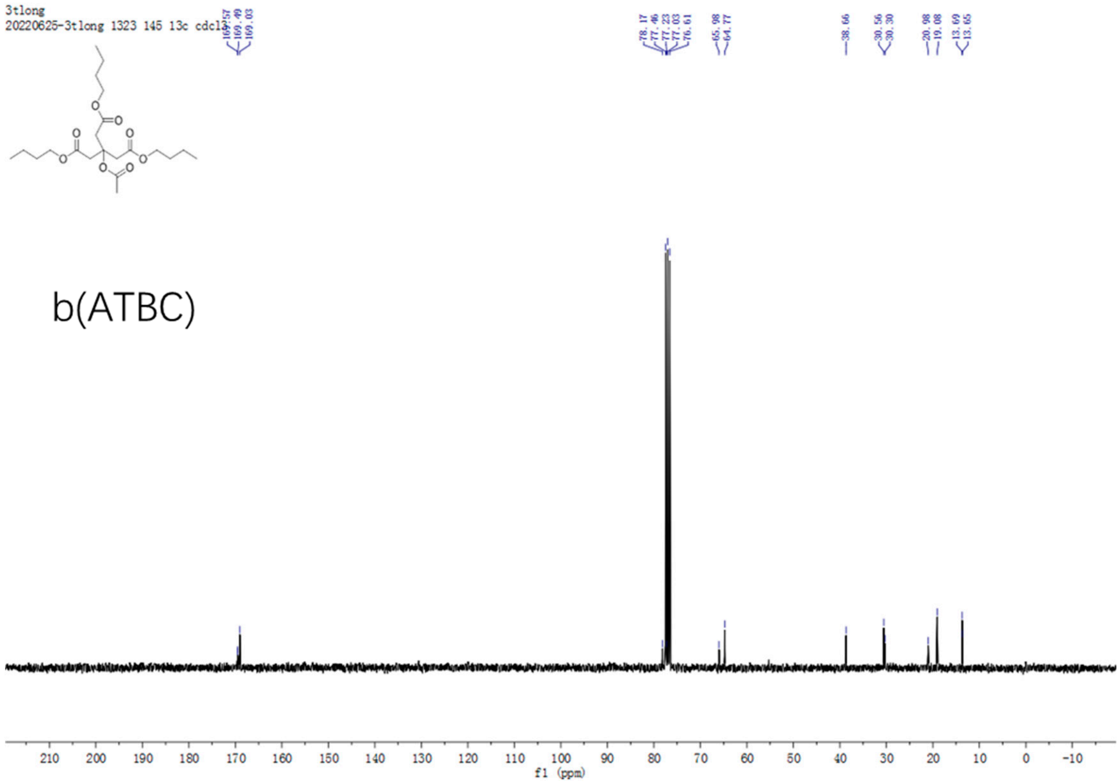
3tlong
20220625-3tlong 1323 145 13c
169.49
169.03
78.17
77.46
77.23
77.03
76.61
65.98
64.77
38.66
30.56
30.30
20.98
19.08
13.69
13.65
b(ATBC)
f1 (ppm)

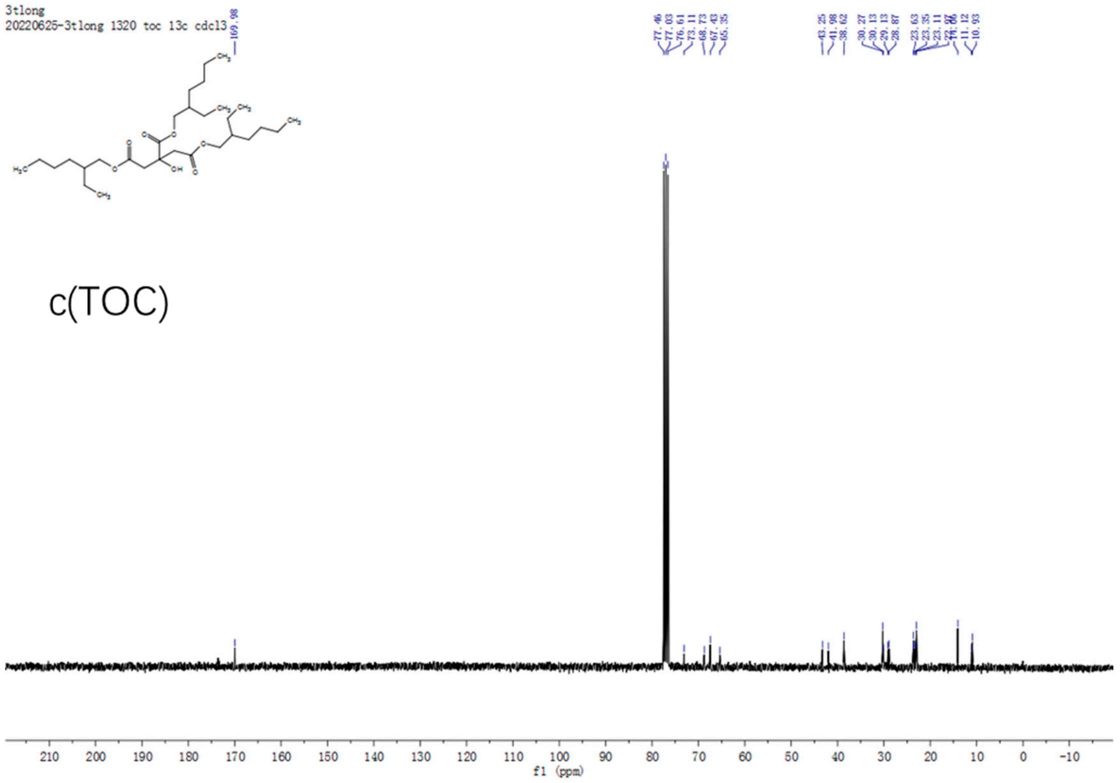

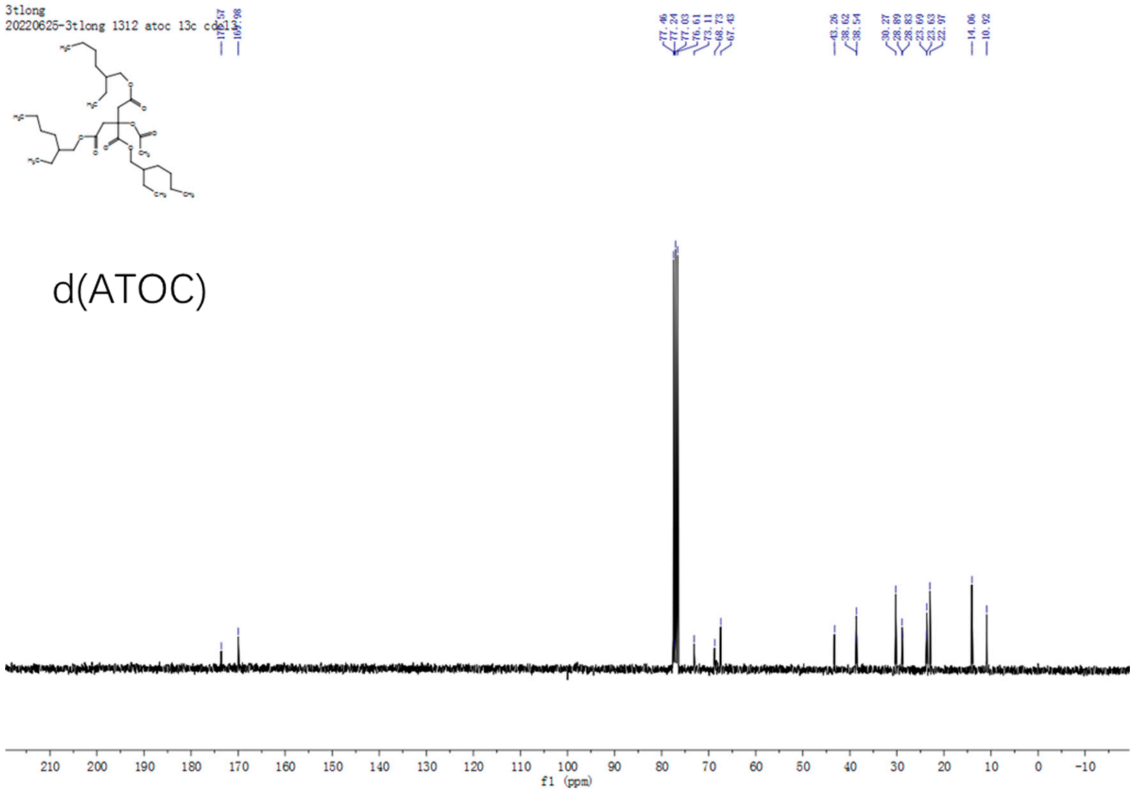

Figure S14. ^{13}C-NMR spectra of products TBC, ATBC, TOC, and ATOC.(a). The 13C NMR (75 MHz, $CDCl_3$) values were δ 178.50, 169.89, 73.20, 66.22, 64.91, 43.33, 30.51, 30.41, 19.07, and 13.68. (b). The 13C NMR (75 MHz, $CDCl_3$) values were δ 169.57, 169.49, 169.03, 85.98, 84.77, 38.66, 30.56, 30.30, 20.98, 19.08, 13.69, and 13.65. (c). The 13C NMR (75 MHz, $CDCl_3$) values were δ 169.98, 73.11, 68.73, 67.43, 65.35, 43.25, 41.98, 38.62, 30.27, 30.13, 29.13, 28.87, 23.63, 23.35, 23.11, 22.97, 14.06, 11.12, and 10.93. (d). The 13C NMR (75 MHz, $CDCl_3$) values were δ 175.57, 165.98, 73.11, 68.73, 67.43, 43.26, 38.62, 38.54, 30.27, 28.89, 28.83, 23.69, 23.63, 22.97, 14.06, and 10.92.

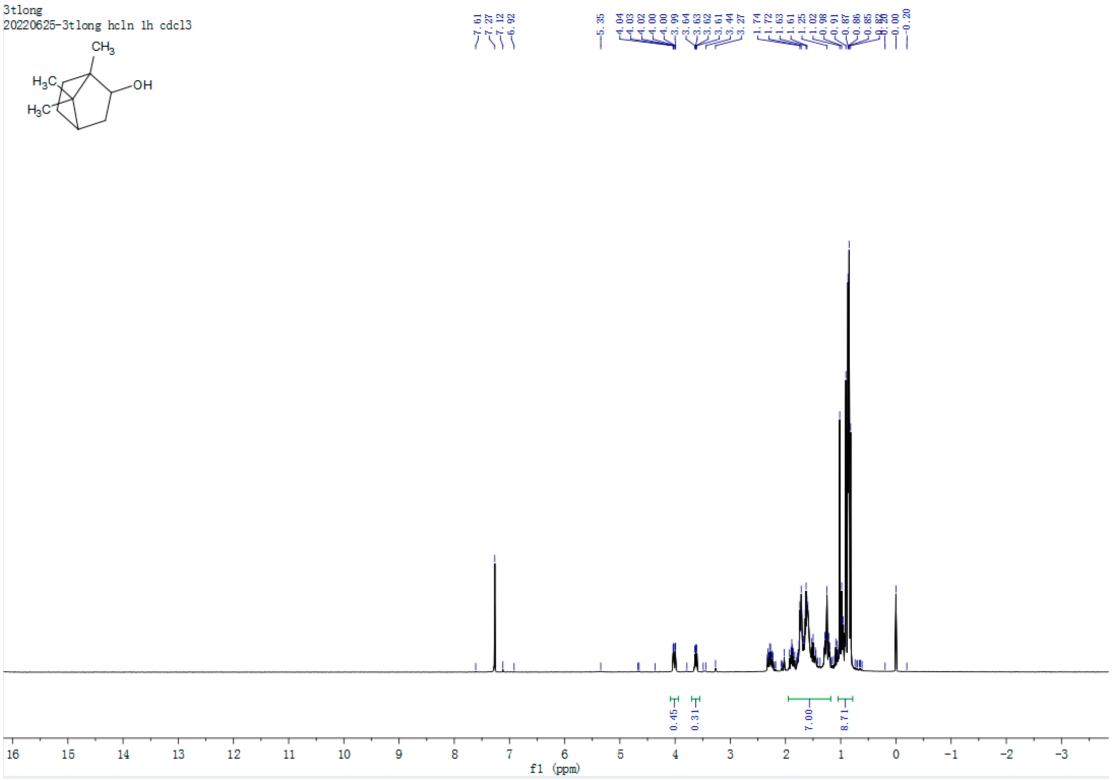

Figure S15. ^{1}H-NMR spectra of borneol

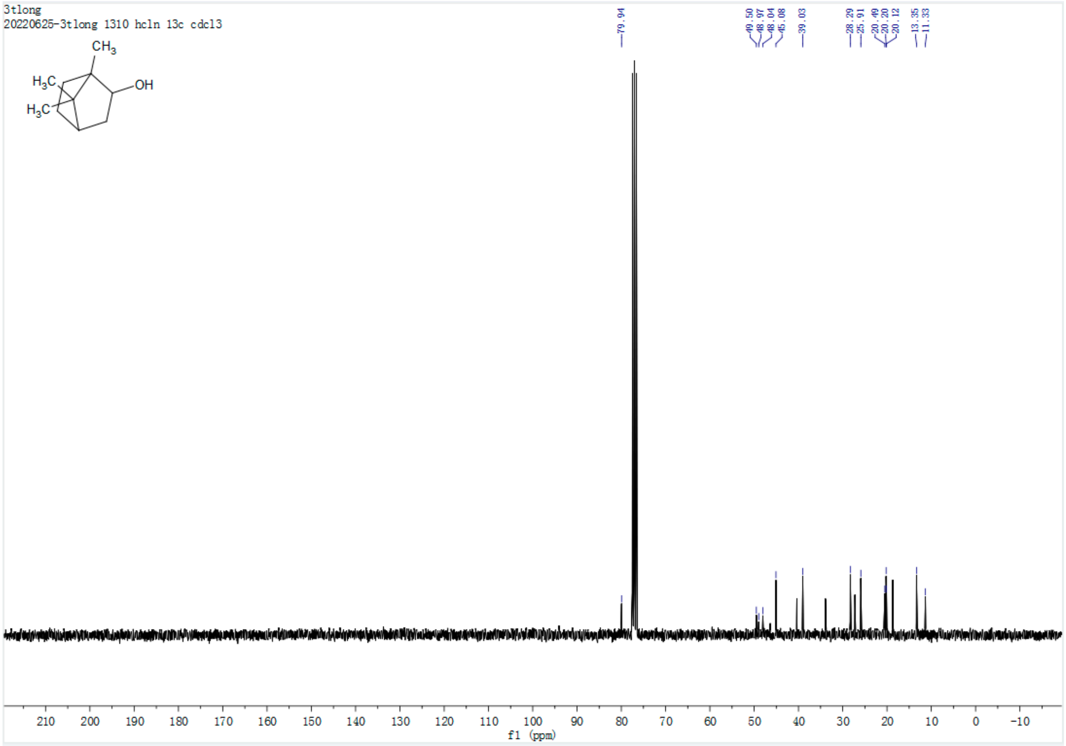

Figure S16. ^{13}C-NMR spectra of borneol
